# Annotations

**Published:** 1895-11-30

**Authors:** 


### ANNOTATIONS.

### The Chemical Sterilisation of Water.

The conclusions arrived at by Dr. Bassenge, in regard to the possibility of producing a sterile drinking water from infected sources by means of chloride of lime, seem of very great importance, and certainly deserve to be carefully reinvestigated, with a view to determine how far they are reliable; for if they should turn out to be correct they will provide a method of sterilisation of water much more potent than filters, and far more portable than tea kettles. According to *Public Health* this method consists of the addition to each litre of water of about one-tenth of a gramme of active chlorine, corresponding to about 0·15 of a gramme of ordinary chloride of lime. This is sufficient, in about ten minutes, to render the water absolutely sterile, although it may before have been very strongly charged with pathogenic organisms. Any excess of chlorine can be removed by the addition of a little bi-sulphite of calcium, which throws down a slight precipitate of sulphate of lime. The water so treated is stated to be harmless, to have no perceptible taste, and to be capable of being used for a long period without exerting any deleterious effect upon the health, as after the treatment it contains no constituents other than those found in ordinary drinking water. The method may be put into practice thus: Put as much chloride of lime as will lie on the end of a small knife (about one gramme) into five litres of the water in question. Shake it up well, and let it stand for from twelve to fifteen minutes. Then add, drop by drop, solution of bi-sulphite of calcium until there is neither taste nor smell of chlorine. It is hoped that this method will be of great use in small surveying expeditions in tropical regions, in encampments of troops, and in small settlements; but it is clear that if it should turn out to be efficacious it will prove of service in endless cases in which, for one cause or another, suspicion lies on the water supply.

### Salvation Army Shelters.

The decision which was given on Thursday at the Southwark Police Court, in the case against the Salvation Army for overcrowding one of their shelters, is of considerable interest, showing, as it does, that wrong may not be done even under the cloak of charity. It is a distressing thing that every night there should be such numbers of people left in such straits of poverty and wretchedness that they are glad to accept the benefit of a shelter from the weather, even if it involve the necessity of sitting on a bench all night, and we may express the highest admiration of an organisation like that of the Salvation Army, which does what it can to shelter these homeless ones. At the same time, this shifting of public responsibilities on to the shoulders of private individuals or charitable associations is a questionable policy, and it is impossible not to see that this decision, just in proportion as it makes it difficult for private charity to deal with this perennial difficulty of homelessness, throws all the more urgently upon the public authorities the duty and the obligation of making some much more efficient provision than exists at present for dealing with those who find themselves at times unable to procure even a lodging. The casual ward does not meet the difficulty, the municipal lodging-house, so far as its development has gone at present, is beyond the means of the people who are now in question. Something intermediate is required, and this the Salvation Army tries to give. Probably the accommodation offered was poor, possibly health even was risked in accepting it, but at least it was a shelter from the weather, and as such was gladly accepted. Now, however, that the vestry has succeeded in limiting the shelter so given, it lies with the vestry to provide some other form of refuge. In Berlin it is found possible to give a bunk, a covering, a bath, some food, and a complete disinfection of the clothes, for about a penny a night, and if this is done by the municipality there why should it not be done by the vestry or the County Council here? The vestries have in the present case made much of the danger to public health likely to arise from the aggregation of these poor people, and there is every reason to believe that so far as public health is concerned it would pay the sanitary authorities to give these homeless ones both food and lodging free, on condition of being allowed to bathe them and disinfect their clothing. Infectious maladies are largely carried about by tramps and by the wandering homeless poor. If the vestries are really in earnest let them see to it that these people are disinfected and rendered harmless, even though it be at the expense of a free bed and a free breakfast.

### The Wholesale Poisoners of London.

The poisoning which is done from such motives as are supposed to influence the poisoner of the novelist is as nothing compared with the poisoning which is attempted to be done every day by those whose one motive is the making of a few shillings or pounds of extra profit in business transactions. Dr. Sedgwick Saunders, the well-known Medical Officer of Health for the City of London, gives, in his annual report for 1894, some very startling statistics on this subject. The people who are so ready to poison unoffending Londoners on such a tremendous scale are often our very innocent-looking country cousins. These gentlemen, aided and abetted no doubt by their metropolitan "friends," sent up last year no less than 430 tons of diseased meat; that is, excluding Sundays, about a ton and a half for every working day in the year. Now a ton and a half of diseased and putrid meat reduced to pounds consists of 3,360, and as each pound is amply sufficient to poison its man, woman, or child, it follows that our dearly-beloved cousins in the country are willing to poison us Londoners to the tune of 3,360 per diem, or, excluding Sundays, at the rate of 1,051,680 per annum. In other words, if we were to actually eat all the diseased meat which our own and other countries send to us, it would not take more than four or five years to accomplish the poisoning of every man, woman, and child in London. This is, perhaps, rather an exaggerated way of looking at the question. Nevertheless, that even this presentment of the case does not go beyond its possibilities is certain; and it is well for us sometimes to see the worst possibilities in order that we may recognise the full measure of our indebtedness to science and civilisation. What becomes of this enormous mass of diseased and putrid fish and meat? The question is an anxious one. Happily Dr. Sedgwick Saunders has it dealt with in a thoroughly scientific way. He immerses it all for several hours in a bath of chloride of sodium, chloride of calcium, sulphate of iron, picric acid, and water. When it has been thoroughly immersed in this fluid for awhile no living creature, human being or lower animal, would look at it as food. It is destroyed effectually for any purpose except of the agriculturist or manufacturer of chemicals.

